# Italian Case Report with a Double Mutation in PSEN1 (K311R and E318G)

**DOI:** 10.3390/neurolint14020034

**Published:** 2022-05-16

**Authors:** Paola Bisceglia, Filomena Lo Vecchio, Raffaela Rita Latino, Carolina Gravina, Maria Urbano, Annamaria la Torre, Gaetano Desina, Antonio Greco, Maurizio Leone, Annibale Antonioni

**Affiliations:** 1 Laboratory of Gerontology and Geriatrics, Fondazione IRCCS Casa Sollievo della Sofferenza, 71013 San Giovanni Rotondo, Italy; p.bisceglia@operapadrepio.it (P.B.); f.lovecchio@operapadrepio.it (F.L.V.); cgravina@operapadrepio.it (C.G.); m.urbano@operapadrepio.it (M.U.); a.latorre@operapadrepio.it (A.l.T.); a.greco@operapadrepio.it (A.G.); 2 Neurology Unit, Fondazione IRCCS Casa Sollievo della Sofferenza, 71013 San Giovanni Rotondo, Italy; latinoraffaelarita@gmail.com (R.R.L.); m.leone@operapadrepio.it (M.L.); 3 Clinical Laboratory Analysis and Transfusional Medicine, Fondazione IRCCS Casa Sollievo della Sofferenza, 71013 San Giovanni Rotondo, Italy; g.desina@operapadrepio.it; 4 Unit of Clinical Neurology, Department of Neuroscience and Rehabilitation, S. Anna University Hospital, 44124 Ferrara, Italy

**Keywords:** Alzheimer’s disease, mild cognitive impairment, *PSEN1*, apolipoprotein E, beta-amyloid

## Abstract

Alzheimer’s disease (AD) is the most common cause of dementia worldwide. The clinical spectrum of suspected AD has been extended from mild cognitive impairment (MCI) to preclinical AD which includes people who have typical cognitive function but harbor the underlying biological features of AD. We report the first case of an Italian patient affected by MCI (MMSE 24\30), characterized by a double mutation p.Lys311Arg (K311R) and p.Glu318Gly (E318G) in Presenilin-1 but with the absence of abnormal accumulation of amyloid beta.

## 1. Introduction

Alzheimer’s disease (AD) is a progressive, irreversible neurodegenerative disorder that is characterized by a decline in memory, language, problem-solving and other cognitive abilities. It ranges in degree from mild to severe which can compromise the activities of daily life [1,2]. AD is characterized by the presence of amyloid plaques (sometimes called senile plaques) formed by misfolded and aggregated Aβ in the extra-cellular zone, and tau-based neurofibrillary tangles (NFTs) [3] intracellularly, which may cause neuronal dysfunction and death. AD brains are further characterized by severe synaptic and neuronal loss as well as reactive astrogliosis and microgliosis [4]. The pathophysiology of AD is still largely unknown and arises from a complex interplay between genetic and environmental factors. The scientific literature has identified MCI (mild cognitive impairment) as a clinical condition that is characterized by a nuanced difficulty in one or more cognitive domains (such as memory, attention or language) and objectified by neuropsychological tests, but without normal and daily activities being compromised. In a non-negligible percentage, MCI patients develop a definite AD every year. Consequently, considerable efforts have been made to better characterize this condition and to identify the mechanisms that explain the evolution to dementia [5]. Most patients with AD are affected by sporadic forms of the disease but few cases are caused by genetic mutations in the amyloid protein precursor (*APP*, located in the chromosome region 21q21.2) and the presenilin genes (*PSEN1*, located in the chromosome region 14q24.3, and *PSEN2*, located in the chromosome region 1q42.13). The diagnosis criteria of AD are evolving rapidly, and the identification of pathogenic mutations is still of vital importance in the diagnosis of AD. Nevertheless, although these three causative genes have been widely investigated, the interpretations of variants remain complex [6].

These mutations, identified in familial AD (FAD), alter *APP* and lead to the overproduction of a longer, toxic version of Aβ42. The *APOE* gene (located at 19q13.2) encodes a glycoprotein (34kDa) that is composed of 299 amino acids (apoE) and is associated with low-density lipoproteins (LDL), very-low-density lipoproteins VLDL and high-density lipoproteins (HDL) [7]. In the CNS, ApoE mediates the neuronal delivery of cholesterol which is an essential component for axonal growth, synaptic formation and remodeling events that are crucial for learning, memory formation and neuronal repair. The *APOE* gene is a polymorphic gene which contains two single nucleotide polymorphisms (SNPs): rs429358 and rs7412. These polymorphisms are responsible for the three major variants, ε2, ε3, and ε4. The ε2 allele is associated with the lowest late-onset AD (LOAD) risk, whereas the ε4 allele is associated with an increased risk for LOAD and a younger age-of-onset of dementia in a dose-dependent manner [8,9]. Thus, the greatest risk factors for LOAD are old age [10,11], carrying the ε4 form of the *APOE* gene [12] and having a family history of Alzheimer’s Disease [13]. The definitive diagnosis of AD requires post-mortem evaluation of the brain tissue. In living patients, cerebrospinal fluid (CSF) and positron emission tomography (PET) biomarkers, combined with several relatively new clinical criteria can help reach the diagnosis [14]. Although genetic forms are a minority among AD patients, the research highlighted the presence of patients with clinical forms of the disease who were suffering from previously unknown genetic mutations. These mutations, in addition to providing support to further understand the pathophysiological mechanisms of AD, allowed the study of non-conventional forms of AD with regard to CSF and instrumental investigations (i.e., PET, Brain MRI). Here, we describe the case of a patient suffering from a mild form of cognitive decline, with absence of pathological findings in CSF and PET but carrying a double mutation in *PSEN1* that is rarely diagnosed in patients with AD. Therefore, this case report aims to deepen the knowledge of the genetic mechanisms responsible for AD and to describe the atypical findings documented in this case report with traditional methods of investigation. 

## 2. Materials and Methods

Genomic DNA was purified from fresh blood samples by the Salting-out method [15]. PCR was performed with 50 ng of genomic DNA, 10 pmol of each primer and 1U of Platinum II Taq Hot-Start DNA Polymerase (ThermoFisher Scientific, Somerset, NJ, USA) in the following conditions: 2 min at 94 °C, followed by 30 cycles: 98 °C (5 s), 60 °C (15 s). PCR products were purified with ExoSAP-IT (Affymetrix, Santa Clara, CA, USA) and sequenced in both directions using a BigDye terminator v1.1 chemistry on an ABI PRISM 3130XL Genetic Analyzer (Applied Biosystems, Austin, TX, USA). The PCR product from the mutated sample was cloned into a TOPO^TM^ TA Cloning^TM^ Kit with a pCR^TM^ 2.1-TOPO^TM^ (ThermoFisher Scientific, Somerset, NJ, USA), and the recombinant plasmid was used for transforming into DH5α Competent Cells (ThermoFisher Scientific, Somerset, NJ, USA), according to the manufacturer’s instructions. Twenty selected clones were used directly as templates for the PCR with the following primers: 5′-TGTGTGTCCAGTGCTTACCTG-3′ and 5′-TGTTAGCTTATAACAGTGACCCTG-3′ (forward and reverse, respectively). PCR reagents and cycling conditions were the same as indicated above. The amplicons were subjected to Sanger sequencing, as indicated above. Human β Amyloid (1-42) ELISA Kit Wako (Osaka, Japan) was used for the quantitative determination/detection Aβ (1-42) in the CFS sample as a diagnostic marker of AD. The sample was diluted with the Standard Diluent present in the Kit. The suggested dilution was 50-fold. Then the sample was measured.

## 3. Case Presentation

**Figure 1:**
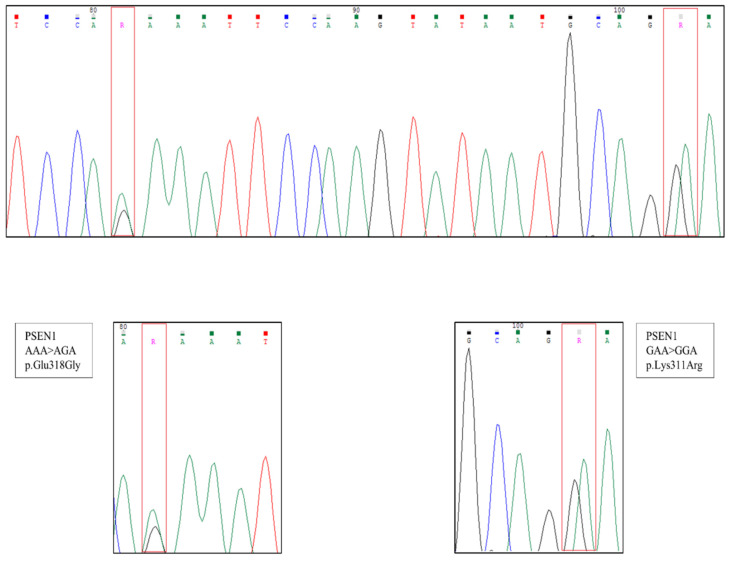
Result from the genomic DNA sequencing revealing a double mutation in PSEN1.

**Figure 2:**
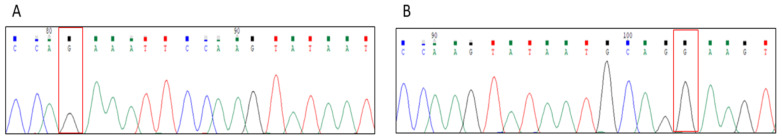
Example of the sequencing results of the molecular cloning performed to determine the cis-trans nature of the variants in the *PSEN1* gene. Twelve clones with p.Lys311Arg (K311R) (**A**) and seven clones with p.Glu318Gly (E318G) (**B**) were identified.

The patient was a 67-year-old man who was married for 40 years and father to two healthy sons. His past medical history was not significant except for a depressive syndrome that began three years before admission and arterial hypertension treated with Zantipress at 30 mg. In September 2018, because of the recent onset of mild memory disturbances, difficulty concentrating and personality changes, he was admitted to the Neurology Unit of the Foundation IRCCS “Casa Sollievo della Sofferenza”, San Giovanni Rotondo, Italy. Memory and concentration deficits were documented through clinical and neuropsychological evaluations (memory deficit, MMSE 24/30). The instrumental investigations (brain CT and MRI) were normal. In May 2019, due to the persistence of symptoms, he was hospitalized again and underwent a lumbar puncture (CSF Aβ _(1-42)_ levels: 742.46 pg/mL; T-tau levels 160.86 pg/mL), (Aβ _(1-42)_ negative if < 650 pg/mL, T-tau negative if values < 350 pg/mL). PET with 18 F-flutemetamol showed no abnormal accumulation of amyloid beta, and an 18-FDG PET scan did not document the presence of significant hypometabolisms in the brain regions of interest. The patient was discharged with a diagnosis of mild MCI in a patient carrying a genetic mutation. Genetic analysis revealed the presence of a heterozygous genetic variant in the *PSEN1* gene (Figure 1), but *PSEN2* or *APP* mutations were not detected. The *APOE* genotype of the patient was ε3/ε4. The molecular cloning technique followed by Sanger sequencing allowed the identification of the trans relationship of genetic variants. Specifically, 20 clones with rs115865530 and seven clones with rs17125721 (Figure 2) were identified. In October 2020, a brain MRI documented some point-like areas of altered signal in the white matter of the semioval centers and in the periventricular site with chronic hypoxic-vascular pathogenesis, and a ventricular system somewhat dilated due to subcortical atrophy. The follow-up documented difficulty in recalling memory, praxic abilities and visuospatial skills. During the visit, his wife reported that he had memory deficits—particularly, memorizing and recalling recently acquired information. The neuropsychological examination, specifically the Mini-Mental State Examination (MMSE), documented a raw score of 27, a corrected score for age and schooling equal to 25, cut-off equal to 24; the Montreal Cognitive Assessment (MoCA) correct score was 21.96 with an equivalent score equal to 3, suggestive of normality. The Brief Psychiatric Rating Scale (BPRS) was administered, and the score was 21. In conclusion, the patient appeared to be suffering from mild cognitive impairment (MCI) with behavioral alterations.

## 4. Discussion

We found the *PSEN1* E318G (rs17125721) and the K311R (rs115865530) mutations in a 67-year-old Italian man with MCI. The *PSEN1* gene is located on chromosome 14 (14q24.3) and it is the most frequently mutated gene in familial AD. The mutations were detected by the direct sequencing of *PSEN1* exon 9. The p.Lys311Arg (K311R) missense mutation was located in the C-terminal cytoplasmic domain in correspondence with the hydrophilic loop of the *PSEN1* gene. This contributes to the pathogenesis of AD by inducing an overproduction of toxic Aβ species (Aβ-42), thus enhancing tau phosphorylation and reducing Aβ-40 levels [16]. Lysine and arginine are basic and hydrophilic amino acids. Thus, the change of lysine into arginine might determine an alteration of the protein function. Despite the presence of the K311R variant, the patient did not show any alteration in the T-Tau CSF levels because there were still no significant correlations in this regard. The variant’s pathogenicity under examination is still of uncertain significance, therefore it will be advisable to wait for the patient’s follow-up to re-evaluate T-tau levels as the disease progresses. The p.Glu318Gly (E318G) was located between two of the eight transmembrane domains of *PSEN1*—specifically, TM7 and TM8, in a large loop domain of *PSEN1*. Through the molecular structure modelling of native *PSEN1* Glu318 and mutated *PSEN1* Gly318, the mutated residue can induce structural alterations in the protein plane through an important torsion of the coil secondary structure. While glutamic acid is a polar, negatively charged amino acid and has an extended flexible side chain, glycine is a nonpolar amino acid and is very small. The change of glutamic acid into glycine can contribute to a decrease in the protein solubility and in the interaction with polar molecules. Furthermore, it can favor the structure folding into a larger and extended form which enables sequence misfolding [17]. The E318G variant is strongly associated with elevated levels of tau and phosphorylated tau in the cerebrospinal fluid (CSF) which suggests that it is an AD risk modifier.[18]. These associations can be an important indicator of a preclinical AD phenotype. However, it is important to underline the presence of recent studies of CSF Aβ _(1-42)_, T-tau and P-tau181 in asymptomatic p.E318G carriers of a large LOAD Italian family that did not reveal any changes in CSF markers [19].

The interaction between *PSEN1* E318G and *APOE* has an important function in the genetic pathways of AD since the carriers of rs17125721 and *APOE*ε4 have an increased risk of developing AD [20]. E318G carriers which are also heterozygous carriers of the *APOE*ε4 allele present a similar AD risk to that of homozygous individuals for *APOE*ε4, and twice the risk of heterozygous individuals for *APOE*ε4 without the E318G allele. The *APOE* genotype was also determined, and this patient was *APOEε3*/ε4. A meta-analysis of clinical studies demonstrated that, compared to individuals with an ε3/ε3 genotype, the risk of AD was higher in individuals with one copy of the ε4 allele (ε2/ε4; ε3/ε4) or two copies (ε4/ε4) [21]. The interaction between ApoE and Aβ has a significant role in the pathogenesis of AD. ApoE may function as an Aβ-binding protein that induces a pathological β-sheet conformational change in Aβ. *APOE*ε4 probably increases the risk of AD by starting and accelerating Aβ accumulation, aggregation and deposition in the brain [21]. The presence of this allele is associated with an increased risk for both early-onset AD and LOAD [22]. With the aim of overcoming the objective limitation of carrying out genetic analyses on the patient’s parents, a study on molecular cloning followed by Sanger sequencing on the proband’s DNA was performed to determine the *cis-trans* relationship of genetic variants. A *trans* relationship between the mutations was defined and it was ascertained that the patient had two alleles, one with p.Lys311Arg (K311R) and another with p.Glu318Gly (E318G). Given the inaccessibility to the patient’s family history, it was impossible to understand the phenotypic consequence of the *trans* nature of the variants, but, in terms of genotypical features, there was a higher probability that the proband had inherited one copy of a mutated gene from each parent and a much lower probability that a case of de novo mutation had occurred.

## 5. Conclusions

Our work represents a further contribution to the literature on MCI, a condition recognized as a possible prodromal phase of subsequent onset of AD, with rare genetic mutations associated with phenotypes with atypical clinical, laboratory and imaging characteristics. This may help to better understand the pathogenetic mechanisms that lead to the onset and progression of the accumulations of pathological materials in this severe and disabling disease, and to develop ideas for new possible therapeutic targets.

## Data Availability

Not applicable.
